# Editorial: Symposium “Pre-results review”

**DOI:** 10.1007/s10683-023-09793-y

**Published:** 2023-02-27

**Authors:** Urs Fischbacher, Irenaeus Wolff

**Affiliations:** 1 University of Konstanz, Universitätsstraße 10, 78457 Konstanz, Germany; 2 Thurgau Institute of Economics, Hafenstrasse 6, 8280 Kreuzlingen, Switzerland; 3 CESIFO, Munich, Germany

Recently, there has been a growing concern with the (non-)replicability of scientific findings. Many reasons for the ‘replication crisis’ have been brought forward, including uncorrected multiple-hypotheses testing, ex-post theorising, p-hacking, publication bias, and under-powered studies (see, for example, the excellent discussion in Renkewitz & Keiner, 2019). Four years ago, *Experimental Economics* called for proposals to be reviewed before collecting data. The aim was to gain experience with this new form of organising the research process that has been gaining popularity in many other disciplines over the past decade.^1^

Reviewing experimental papers before the data has been collected—or pre-results review, for short—has the advantage of addressing many, if not all, of the above problems. Doing so already in the process is particularly important because detecting biases by meta-analytic tools often will be impossible (Renkewitz & Keiner, 2019).^2^ In particular, pre-results review addresses what we see as the most central problem, publication bias. Pre-results review addresses publication bias in two main ways: it incentivises authors to write up experiments with null results and precludes referees and editors from sorting out experiments on important questions just because the answer was negative. We will be more explicit on the benefits below, but for now, let us quote the RR-factsheet-for-editors.pdf (Version: 4) of the Center for Open Science (https://osf.io/jbeus/):

By reducing various forms of bias, it is likely that Registered Reports [i.e., papers selected through pre-results review, addition of the authors] will produce more “negative” results, but a stringent selection at Stage 1 [the pre-results review phase] will ensure that the published results, whether positive or negative, will be among the most credible in the journal. One might also suppose that a relative increase in negative results could lead to lower citation rates. However, since Registered Reports were launched in 2013 they have been highly cited, at average rates exceeding the impact factors of the journals in which they are published.^3^

The Symposium “Pre-Results Review” was envisioned to consist of a collection of papers in an issue of *Experimental Economics* in late 2021. The call was issued in late April 2019, asking for submissions by 15th January, 2020. A submission would consist of a full paper except for the data, i.e. detailing the motivation, related literature, power analysis, specifics of the data analysis and even short ‘contingent’ conclusions for each foreseeable outcome. This submission format does not preclude exploratory data analysis. The only requirement is that any exploratory data analysis is marked as such in the final paper.

By the deadline, 21 submissions had arrived. To put the above number into perspective, note that the *Journal of Development Economics* received 46 submissions in the first 16 months of offering a pre-results review submission option (Foster et al., 2019).^4^ The range of topics for the Symposium was very broad, including the effects of sexual arousal, a dual-process explanation for herding, auctions, bargaining, and corruption.

The 21 submissions were handled by four editors, Urs Fischbacher (10), Lorenz Goette (1), Charles Noussair (1), and Irenaeus Wolff (9). We involved additional editors to avoid handling papers of former or current co-authors. Finding reviewers was reasonable: slightly more than half of all contacted scholars wrote a report. Also, the reviewers’ overall recommendations seem not to have differed substantially from the reviewers’ recommendations on standard submissions. 37 out of 45 reports (82%) recommended rejecting the proposal. We invited resubmissions in three cases, translating into a rejection rate of 86%, which is the same as the current rejection rate of regular submissions of 86% (year 2021; for comparison: the *Journal of Development Economics* had a rate of 16.7% of in-principle accepted papers amongst the fully adjudicated submissions by July 2019, Foster et al., 2019; this rate has not changed since, Foster, personal communication). The authors of one of the three invited revisions decided not to continue with the project along the lines of the proposed changes.^5^ Hence, we are left with a comparatively small Symposium consisting of only two papers.

In the end, the two papers that made it both present new experimental measures. Alós-Ferrer & Granić (2023, this volume) use a new experimental design to test for the economic relevance of the mere-choice effect in the domain of risk-taking. The paper shows a null effect, i.e., the mere-choice effect does not seem to be a concern for economics. Such a null result might be difficult to get published under the standard submission procedure. Bruttel et al. (2023, this volume) show that attitudes towards *strategic* uncertainty depend on the context. Under strategic complementarity, the majority of participants tend to be pessimistic regarding the desired outcome, while strategic substitutability tends to lead to optimism. Given the positive findings, the authors most likely would have had an easier time to get their paper published under the standard submission procedure, compared to the first paper.

## The motivations for introducing pre-results review

As we pointed out above, pre-results review is meant to tackle various forms of questionable research practices. Having said this, our primary motivation for promoting pre-results review is to fight publication bias, which we consider the prime reason for replication issues in psychology and economics. In our view, far too many studies end up in the ‘file drawer’. These studies are not written up due to a lack of incentives because the (perceived) chances of publishing a null result or even inconclusive results are just too low. However, coming back to the above argument, if a stringent selection is applied during the pre-results review phase, the published results, positive or negative, will be among the most credible in the journal. And because the profession rightly is moving away from the idea that one study is generally enough to decide a question, even high-powered inconclusive results are important if the question is important enough.

Widespread usage of pre-results review would have positive side effects. In particular, it would lead to a better-targeted use of resources. First, eliminating the publication bias would strongly reduce the multiplication of efforts of multiple researchers trying to demonstrate a non-existent relationship. Second, false-positive results would become rarer because power calculations are required in Stage 1, which are also reviewed. Consequently, the number of follow-up studies based on false-positive results would be reduced, too. Third, there would be fewer experiments with flawed designs because referees (and sometimes potentially even the authors themselves) would spot bad design choices before the experiments are run. Fourth, acquiring funds may become easier for young researchers if they demonstrate to a funding organisation that the research will be published.^6^ Fifth, while the up-front investment needed for starting the project will increase strongly, obviating the need to re-write a study time and again is likely to decrease overall time costs. In Chris Chambers’ words: “Because the study is accepted in advance, the incentives for authors change from producing the most beautiful story to the most accurate one.”^7^ In this context, the “most accurate” story means a neutral presentation of the data rather than a selection of analyses to fit a particular story. Sixth, in some cases, the procedure may even lead to a wiser choice of projects to be pursued because researchers think about a project more carefully before they start it, and the higher up-front costs are likely to deter them from working on less important research questions. Seventh, as Dufwenberg & Martinsson (2019) have pointed out, even the incentives to cheat are reduced. The basic argument is that if the article will be published irrespective of the results, there is a lower incentive to report particular outcomes and less of a need to fabricate a perfect story. Dufwenberg & Martinsson suggest results-free review exactly as a solution to the problem of existing incentives to cheat. And eighth, pre-results review would allow going back to a meaningful double-blind process for the initial submission, which might attenuate or even eliminate a seniority bias.

## Common reservations and our experience

Having pointed out the advantages and benefits pre-results review has, we now also discuss potential downsides. In the following, we present the most common reservations we have heard and juxtapose them with our experiences where relevant.

The first reservation is that we might receive only low-quality submissions because people might prefer not to submit ‘revolutionary’ ideas. They would like first to conduct the study and decide on the submission target when they can judge the scope of the result. Two things can be said about this first reservation before we look at the data. On the one hand, the up-front costs increase, which should discourage low-quality ideas. On the other hand, for revolutionary ideas, authors may hope to publish the study in a top general-interest journal, which may indeed discourage high-quality submissions. Yet, it is not clear why a discouragement should happen even under the somewhat restrictive terms of the Symposium (that authors cannot hand in the final paper at another journal and fall back on the in-principle acceptance at *Experimental Economics* after a rejection).^8^ Under the published terms, it would have been possible to submit, run the experiment, and re-tract the study for the Symposium in case of revolutionary findings. If authors were to follow this path in substantial numbers, this would indeed be a problem for *Experimental Economics* as a journal, as we would be bearing the costs while another journal would be reaping the benefits. Yet, for science as a whole, the pre-results-review procedure would be beneficial, as it would ensure that the results would be published irrespective of the results. The downside would be that the editors and referees whose time and effort were used to advance science would likely become resentful that they put in hard work as long as they cannot get any credit for it.^9^

Judging by the submissions and the associated reviewer recommendations, there is little evidence of low-quality submissions. Twenty-one submissions is a substantial number in light of the window for submissions. Judging by the recommendations, the submissions had a quality comparable to those of standard submissions to *Experimental Economics*. At the same time, note that Soderberg et al. (2021) ran an experiment in which 353 scientists rated a sample of published, partially blinded publications submitted either under the standard procedure or under pre-results review. The participating scientists had to rate the studies on 19 characteristics. Numerically, the publications that had gone through pre-results review outperformed the standard publications on all characteristics, and significantly so for 12 out of 19 characteristics, amongst them methodologic rigour, how much can be learnt from the article, and overall article quality.

Related to the concern that too few high-quality ideas might be submitted is the concern that people might use pre-results review only as a cheap improvement device for their studies. In the case of our special issue, we chose not to have an ‘entry bar’ in terms of desk-rejecting some of the submissions. The main reason for sending out all submissions for review was that we were inexperienced with the format and wanted to give all submissions their best chance of being included in the Symposium. We probably would change this policy if pre-results review was introduced as an additional standard submission option. Having said this, we did not have the impression that any of the submissions were rough sketches in the state of a preliminary draft. Virtually all of the submissions gave testimony of a substantial amount of thought and effort by the authors. Given that, the “improvement device” (if any) is by no means “cheap”.

Suppose a journal offering pre-results review were to note at some point in time that a non-negligible share of authors is using pre-results review as a cheap improvement device. In that case, one would, of course, need to think of measures that could be taken to address this problem. A first measure would be to introduce desk rejections, as pointed out above. A second measure could be to introduce submission fees, although a submission fee would probably need to be very high to serve as an effective entry bar. A measure that would solve the problem would be not to provide feedback for rejected papers. However, such a measure contradicts transparency requirements and leads to inefficiencies because reviewers’ thoughts would get lost. After all, the most relevant cost may be time, which might be particularly high for innovative ideas in the air. However, time costs are already present if we do not aim for the extremely short turn-around times that some general-science journals work with.

A third reservation is that laboratory research does not need pre-results review because one can easily re-run studies. In our view, this is a good argument for why the pre-registration of hypotheses (which is part of the pre-results-review process) may not be as crucial for lab experiments as it is for field experiments. Laboratory experiments, particularly those considered to be important, are naturally replicated in follow-up experiments, and null results in these replications may eventually find their way into the journals (even though it typically takes more resources to correct a published result than to publish a result without precedent). However, the main aim of introducing pre-results review is to address the publication bias *before* a (false-)positive finding has been published. It guarantees that null results are published before so many attempts are made that a “statistically significant” effect is recorded.

A fourth reservation is that scholars might try to cheat. The concern was that scholars might run an experiment, obtain a null result, and submit the paper results-free as a proposal. The proposal gets accepted based on the research question and the design. There are two possibilities: if the submitting scholars report the old data, that runs under fraud. This is something we cannot prevent (similar to, e.g., faking the data under the standard process). If, however, the scholars re-run the experiment (which they have to do anyways in case reviewers suggest any changes to the design), then new evidence concerning this research question is generated.^10^ The important research question has received an answer, which is an important piece of information. As Dufwenberg & Martinsson (2019) argue, pre-results review even *reduces* the incentives to cheat, particularly in reporting incomplete data sets or tweaking the data (see above).

A fifth reservation is the problem of an increased seniority bias. Again, there are several answers. If pre-results submissions were handled under a double-blind protocol, then any seniority bias should be reduced or prevented. Without a double-blind review, it is an empirical question. Of course, we do not have sufficient data to assess the seniority bias statistically. Nevertheless, two out of three Revise-and-Resubmit decisions went to author teams containing scholars ranking in the top 10% of the Repec Ranking (all publications). At the same time, there were only seven such scholars among the 27 submitting authors. In this light, it might be reasonable to switch back to a double-blind review. This is much easier with pre-results-review submission because nobody will publicly post their research proposals before submission. In contrast, authors are often identifiable in standard submissions because working papers are available online.

A sixth reservation is the risk that authors provide low-quality work at the second stage of the process, in the data collection or the data analysis. Generally, the paper should have high quality because quality is ensured in the first submission stage, and the second submission stage ensures that authors will perform well on the promised analyses. Also, authors are likely to provide high-quality work because their reputation is still at stake. As pointed out above, the evidence from other fields gathered by Soderberg et al. (2021) suggests that the quality does not suffer in any dimension and improves in terms of how much can be learnt from the article. However, it is entirely conceivable that there will be less explorative data analysis. While this is an open empirical question, there is a potential trade-off here. By providing an institution that ensures that analyses become more reliable, we lose some handle in incentivising more detailed exploratory analyses.

A seventh reservation is how to roll out pre-results review on a larger scale. This Symposium provides the first evidence of how it works for the journal—what kind of submissions we can expect and how the refereeing process works. As our discussion above shows, we did not encounter major problems and received good quality. However, implementing pre-results review on a larger scale could affect these outcomes. For example, authors, reviewers, and editors involved in the Symposium may have been particularly motivated. There are also specific issues that would arise. First, implementing pre-results review as a standing option would require another editor on the editorial board. It is less evident whether more reviewers will be needed. On the one hand, the number of initial submissions would increase to a certain degree (our experience suggests an estimate of roughly 30 submissions per year). On the other hand, fewer revisions will be the standard in the second stage because most parts of the second-stage submission have already been reviewed (in the case of the two articles in our Symposium, there was no need to re-invite the reviewers in the second stage altogether).^11^

It would definitively be necessary to extend the editorial system to add additional default texts and to take into account the additional states (most prominently, “in-principle accepted”; for the Symposium, we technically had to mark in-principle-accepted submissions as “revise and resubmits”). A larger roll-out also requires—in particular at the beginning—more instructions for editors and referees to explain the specific features of the new reviewing process.

The most prevalent concern, however, is what the above quote already hinted at: that the journal might end up publishing ‘too many uninteresting results’. But then, again, shouldn’t it be more important to us to get the true answer to an important question, examined through a well-suited design, than to read about ‘interesting results’ that have a far higher probability of being false positives? We need to know ‘uninteresting results’ if they are answers to important questions. And pre-results review is the best way to ensure that.

Overall, we are convinced that pre-results review is an option that solves many problems concerning the replication crisis. This Symposium also shows that it is feasible in principle. For *Experimental Economics*, we see two reasons to consider it risky to roll it out at the current stage. First, more experience is needed to know how the process scales up. Second, pre-results review is still uncommon in economics, and so *Experimental Economics* might lose in-principle-accepted papers to top journals.

## Footnotes

1 As of today, there are 280 journals documented on https://www.cos.io/initiatives/registered-reports that use pre-results review as a regular submission option. There are several journals with a high reputation that are also open for submissions from (behavioral) economics, for example Nature Communications, Nature Human Behavior, Evolution and Human Behavior, and the Journal of Development Economics. About half a year ago, the *Journal of the Economic Science Association* also introduced pre-results review, albeit for replication studies only.

2 Renkewitz & Keiner (2019) show that once the true effect is even slightly heterogeneous—because of slight variations between replications of the same study, be it in terms of the protocol, the experimenter, the participant pool, or any other variation—it is no longer possible to detect any bias by the meta-analytic tools that are commonly used.

3 While the question of whether there is a causal relationship in this regard is still open, the evidence in Soderberg et al. (2021) suggests that registered reports may in fact be of higher quality overall (addition by this Symposium’s guest editors).

4 If we assume a linear pattern of submissions, this would translate into roughly 23 submissions within a comparable amount of time, which is very similar to our submission count (after the initial pilot phase and abstracting from a year-long dip due to COVID-19, the *Journal of Development Economics* has had a steady flow of 2 pre-results-review submissions per month; Foster, personal communication).

5 The withdrawal rate of regular submissions equals 2% (year 2021). One out of 21 submissions means a withdrawal rate of 4.8% for the Symposium.

6 This would work in particular if funding agencies cooperate with journals offering pre-results review, as suggested by Chambers & Tzavella (2022). As long as this is not the case, there could also be a danger for the journal if in-principle-accepted submissions have to be retracted because the authors could not obtain fundings.

7 Cited on: https://www.cos.io/initiatives/registered-reports, last accessed on 24th October, 2022.

8 At the *Journal of Development Economics*, authors are free to submit a paper elsewhere and fall back on the in-principle acceptance in case the paper is not accepted at the other outlet(s).

9 A partial solution that was suggested by members of the editorial board is to make submitting authors agree to carrying the paper’s history to publication on any working paper and the final published paper. A footnote on the first page would then include where it was submitted for pre-results review first. Admittedly, this solution is not complete because authors could claim that the final publication is a new paper.

10 Of course, it is scientifically correct and most efficient to report both data sets.

11 If the withdrawal rate of submissions invited for revision and resubmission was as high as in our Symposium—one out of three—much of the additional effort would be futile, of course. However, given that the *Journal of Development Economics* does not seem to have problems of that sort, we would see the withdrawal as an outlier.
